# In the Children's Ward

**Published:** 1887-02-12

**Authors:** Helen Milman


### IN THE CHILDREN'S WARD.

AN angel was passing the length of the ward,
Sent straight from the Saviour's side,
For there in His glory, midst angels, at once
He knew that a little one cried.

The others were sleeping; and only the child
Lay watching, alone, and in pain,
And wond'ring, in childish impatience, "if e'er
The daylight was coming again!"

"If Jesus," she whispered, "could know I was here,
And knew how I try to be good,
I'm sure He would make me get well quick again,—
Oh! yes, I feel *sure* that He would!

"But I think that the angels are singing too loud
For Him to be able to hear;
It cannot be He has forgotten up There!
For He bade little children draw near.

"I'll say all my prayers all over again,
It's because its such *miles* to the sky,
I wish He would carry me up in His arms,
It would be so *lovely* to die!

"They say that I cannot get well, or at least,
Can never be healthy or strong;
So if I beg Jesus to carry me home,
I hope He won't think I am wrong."

And then, as the tears fell in weariness down,
A bright vision appeared to her sight,—
A beautiful angel came gliding along,
On the floor leaving footsteps of light;

And bending right over the little one's cot,
She bade her look skyward, and see
How the angels were peeping thro' stars, as they sang
"Christ hears, and is waiting for thee!"

So morning crept gently all over the ward,
And the first of the sunbeams shone where
A little one sleeps! whose soul is with Him
Who heard, and who *answered her prayer.*

HELEN MILMAN.

